# The Routine Utilization of Dental Care during Pregnancy in Eastern China and the Key Underlying Factors: A Hangzhou City Study

**DOI:** 10.1371/journal.pone.0098780

**Published:** 2014-06-05

**Authors:** Wei Sun, Jing Guo, Xiuyang Li, Yongqi Zhao, Hui Chen, Gang Wu

**Affiliations:** 1 Department of Conservative Dentistry and Periodontics, Affiliated Hospital of Stomatology, Medical College, Zhejiang University, Hangzhou, Zhejiang, P.R. China; 2 Department of Oral Cell Biology, Academic Centre for Dentistry Amsterdam (ACTA), Research Institute MOVE, VU University and University of Amsterdam, Amsterdam, the Netherlands; 3 Department of Epidemiology & Biostatistics, Zhejiang University, Hangzhou, P.R. China; 4 Department of Oral Implantology and Prosthetic Dentistry, Academic Centre for Dentistry Amsterdam (ACTA), Research Institute MOVE, VU University and University of Amsterdam, Amsterdam, the Netherlands; University of Toronto, Canada

## Abstract

**Objectives:**

Oral diseases are associated with adverse pregnancy outcomes. The routine utilization of dental care (RUDC) during pregnancy is an effective way to improve pregnant women’s oral health, and thus safeguard the health of their babies. As China has one fifth of the world’s population, it is especially meaningful to encourage RUDC there. However, the status of RUDC in China and the key underlying factors are largely unknown.

**Methods:**

This cross-sectional survey investigated the current status of RUDC during pregnancy and the key underlying factors in Hangzhou City, Zhejiang Province, eastern China. We collected participants’ demographics, individual oral-hygiene behaviors, individual lifestyle, oral-health conditions and attitudes, and also their RUDC during pregnancy. Binary Logistic Regression Analysis was used to analyze the key underlying factors.

**Results:**

Only 16.70% of the participants reported RUDC during pregnancy. The percentage of RUDC was significantly lower among pregnant women with the following characteristics: aged 30 or less, an annual household income under $8,000, brushing once a day or less, never flossing or rinsing the mouth, paying no attention to pregnancy-related oral-health knowledge, and being dissatisfied with one’s individual dental hygiene behavior.

**Conclusions:**

RUDC during pregnancy is very low in eastern China and is greatly influenced not only by a woman’s age, annual income, individual hygiene behavior, but also by her attention and attitudes to oral health. To improve this population’s access to and use of dental care during pregnancy, appropriate programs and policies are urgently needed.

## Introduction

Pregnancy is a modified physiological state that affects the oral environment, particularly the periodontal tissues. Since the publication of the first international report in 1963 [1], it has been the consensus that pregnant women have a significantly higher incidence of inflammatory response in periodontal tissue than non-pregnant women [2]. Accumulating evidence has also indicated that periodontal disease is an independent risk factor for adverse pregnancy outcomes, such as pre-term birth and low birth-weight babies [3]–[5]. This phenomenon can possibly be attributed to two mechanisms: 1) periodontal disease may affect the maternal and fetal immune responses systemically, thereby leading to premature labor; 2) oral bacteria may translocate directly into the pregnant uterus, causing localized inflammation and adverse pregnancy outcome. Treatment of periodontal diseases is therefore thought to reduce the incidence of adverse pregnancy outcomes–a notion that was supported in a recent clinical trial which found that periodontal treatment completed before the 35^th^ week had a beneficial effect on birth weight and time of delivery [6]. Routine utilization of dental care (RUDC) during pregnancy is thus a pregnant woman’s last opportunity to improve her own oral health and to safeguard her baby’s health more generally.

The advantages of improving of pregnant women’s oral health also lie in the fact that mothers with regular access to dental care are more likely to take their children to the dentist, thereby enabling mother and child alike to develop the attitudes and behaviors that promote oral health [7]. Programs and policies are thus urgently needed to encourage RUDC in pregnant women. One such program in Oregon, USA significantly raised the utilization percentage from 8.8% in 2001 to 55.8% in 2006 [8].

Although such programs and policies are extremely important in China, which has one fifth of the world’s population, the country has established very few programs for pregnant women to improve their dental knowledge and to encourage their RUDC. Neither have there been any systematic surveys to investigate RUDC and the key factors underlying pregnant women’s practice of it–a situation that greatly hampers the establishment of effective policies and programs for improving pregnant women’s oral health.

In this study, we therefore performed a cross-sectional survey to investigate RUDC and the key factors underlying it in Hangzhou, Zhejiang Province, eastern China. We analyzed the influence of 14 factors in four domains: demographics, individual oral-hygiene behaviors, individual lifestyle, and current oral-health conditions and attitude to dental care. We aimed to provide data that would instruct the development of programs and policies for improving women’s oral health, and thereby the health of their babies.

## Methods

### Study Population

The conduct of this study was approved by the Review Board of the Affiliated Hospital of Stomatology, Medical College, Zhejiang University, China. The survey was conducted from December 2010 to May 2011. To request their participation in the study, we approached pregnant women who were receiving pregnancy courses at three important women’s hospitals in Hangzhou: 1) the Women’s Hospital, Medical College, Zhejiang University; 2) the Gongshu District Centers for Disease Prevention and Control and for Women and Children’s Healthcare; and 3) the Jianggan District Center for Women and Children’s Healthcare. All participants signed informed consent. Questionnaires were used to gather information on demographic information, oral health status, and utilization of dental care. As an incentive to complete the questionnaire, each woman was compensated with a gift (a bottle of antiseptic rinses).

### Data Collection

The main dependent variable was RUDC during pregnancy. To investigate the key factors underlying a woman’s decision regarding RUDC, we collected data regarding 14 factors in four domains (Table 1): A) demographics (age, education, annual household income and source of payment for dental care); B) individual oral hygiene behaviors (frequencies of brushing, flossing and mouth-rinsing); C) individual lifestyle (smoking and alcohol-drinking); D) women’s oral-health conditions and their attitudes to dental care.

**Table 1:** Descriptive characteristics of the pregnant women.

Characteristics of pregnant participants (n = 2259)						
Characteristics	Number	Percentage (%) in total participants	Had routine utilization of dental care in each category		Had no routine utilization of dental care in each category	
*A) Demographics*			Number	Percentage	Number	Percentage
*1 Age in years*				(%)		(%)
Under 20	2	0.09	0	0	2	100
21 to 25	242	10.71	20	8.51	215	91.49
26 to 30	1375	60.87	217	16.15	1127	83.85
31 to 35	504	22.31	101	20.95	381	79.05
36 or older	125	5.53	27	22.88	91	77.12
Data missing	11	0.49	1	9.09	10	90.91
*2 Educational attainment*						
Less than a high school diploma	143	6.33	11	8.27	122	91.73
High school diploma	210	9.3	24	11.82	179	88.18
Some college, or a two-yearcollege degree	375	16.6	52	14.21	314	85.79
Four-year college degree	838	37.1	144	17.45	681	82.55
Master degree or higher	587	25.98	113	20.07	450	79.93
Data missing	106	4.69	22	21.57	80	78.43
*3 Annual household income*						
Less than $4.500	453	20.05	42	9.68	392	90.32
$4.500 to $8.000	660	29.22	79	12.46	555	87.54
$8.000 to $16.000	817	36.17	167	20.67	641	79.33
More than $16.000	253	11.2	71	28.86	175	71.14
Data missing	76	3.36	7	10	63	90
*4 Source of payment for dental care*						
Cooperative medical service	47	2.08	7	15.56	38	84.44
Private health insurance	1128	49.93	186	16.80	921	83.20
National insurance	115	5.09	18	17.14	87	82.86
Reimbursement from employers	193	8.54	49	26.20	138	73.80
Others	21	0.93	6	28.57	15	71.43
Pay by themselves	743	32.89	100	13.95	617	86.05
Data missing	12	0.53	0	0	10	100
***B) Individual oral hygiene behaviors***						
*5 Frequency of brushing tooth*						
Never	17	0.75	2	12.50	14	87.50
Once a month	13	0.58	4	36.36	7	63.64
Once a week	11	0.49	1	10.00	9	90.00
Every day	978	43.29	100	10.65	839	89.35
Two or more times per day	1238	54.8	259	21.32	956	78.68
Data missing	2	0.09	0	0	1	100
*6 Frequency of flossing*						
Never	1771	78.4	223	12.95	1499	87.05
Once a month	156	6.91	39	26.35	109	73.65
Once a week	159	7.04	41	26.45	114	73.55
Every day	130	5.75	41	32.03	87	67.97
Two or more times per day	34	1.51	18	58.06	13	41.94
Data missing	9	0.4	4	50	4	50
*7 Frequency of rinsing mouth*						
Never	1347	59.63	171	12.96	1148	87.04
Once a month	193	8.54	44	23.78	141	76.22
Once a week	214	9.47	47	22.49	162	77.51
Every day	338	14.96	64	19.94	257	80.06
Two or more times per day	158	6.99	38	25.00	114	75.00
Data missing	9	0.4	2	33.33	4	66.67
***C) Individual behavior***						
*8 Smoking*						
Before pregnancy	58	2.57	8	14.81	46	85.19
During pregnancy	10	0.44	4	44.44	5	55.56
Never	2183	96.64	354	16.68	1768	83.32
Data missing	8	0.35	0	0	7	100
*9 Drinking alcohol*						
Before pregnancy	575	25.45	92	16.55	464	83.45
During pregnancy	25	1.11	6	26.09	17	73.91
Never	1638	72.51	267	16.72	1330	83.28
Data missing	21	0.93	1	6.25	15	93.75
***D) Participants’ oral health conditions and satisfaction with these conditions***						
*10 Do you pay close attention to pregnancy-related oral-health knowledge?*						
Yes	1402	62.06	302	22.17	1060	77.83
No	833	36.87	60	7.36	755	92.64
Data missing	24	1.06	4	26.67	11	73.33
*11 Do you have swelling and hyperplasia in the gingiva during pregnancy?*						
No	1496	66.22	235	16.18	1217	83.82
In partial gingiva	707	31.3	121	17.54	569	82.46
Most gingival	12	0.53	4	36.36	7	63.64
Data missing	44	1.95	6	15.38	33	84.62
*12 Do you have tooth pain or peri-apical inflammation during pregnancy?*						
No	1772	78.44	284	16.44	1443	83.56
In partial gingiva	363	16.07	59	16.71	294	83.29
Most gingival	82	3.63	17	21.52	62	78.48
Data missing	42	1.86	6	18.18	27	81.82
*13 Are you satisfied with your oral health?*						
Satisfied	788	34.88	148	19.40	615	80.60
not very satisfied	1325	58.65	204	15.85	1083	84.15
completely dissatisfied	142	6.29	13	9.35	126	90.65
Data missing	4	0.18	1	33.33	2	66.67
*14 Are you satisfied with your individual dental hygiene behavior?*						
Satisfied	833	36.87	193	24	611	76
not very satisfied	1325	58.65	167	12.95	1123	87.05
completely dissatisfied	89	3.94	5	5.75	82	94.25
Data missing	12	0.53	1	9.09	10	90.91

These data were collected through self-reported answers to forced-choice questions. In total, we sent out 2500 questionnaires, 2264 of which were completed and returned. After excluding any questionnaires that lacked data for three or more factors, we included 2259 questionnaires for further statistical analysis.

### Statistical Analysis

After summarizing the data from the questionnaires included, we generated Table 1 in order to show the descriptive frequency of each underlying factor. To identify the key factors underlying RUDC, binary logistic regression analysis was used. First, to analyze the association of RUDC with each candidate factor, we performed Univariate analyses. The factors that showed a significant association (*P*<0.10; see Table 2) were considered as candidate factors for the subsequent forward stepwise multivariate logistic regression analyses, which were used to gradually generate the final logistic model (Table 3). Significance was set at *P*<0.05 and the confidence level at 95%. On the basis of the present data, we used the following formula to calculate the sample size needed for this logistic regression: *n* = (Z_α/2_)^2^×π× (1−π)/δ^2^, where π was the percentage of RUDC among the participants. We set α (confidence interval) as 95%; and set δ (admissible error) as 10% π. Then, the needed sample size is 1912 [*n* = (1.96)2×0.1673×(1–0.1673)/0.016732]. Our sample size of 2259 was sufficient for this logistic regression.

**Table 2:** Univariate results per candidate factor.

	Full model	
Characteristics	OR‡ (95% CI§)	P
1 Age		
30 or less	1	
31 or older	1.538(1.211–1.953)	<0.001
2 Educational attainment		
Less than four-year college degree	1	
Four-year college degree or more	1.606(1.236–2.088)	<0.001
3 Annual household income		
Less than $8,000	1	
More than $8,000	2.283(1.799–2.896)	<0.001
4 Source of payment for dental care		
Self-paid	1	
Others	1.369(1.066–1.757)	0.014
5 Frequency of tooth brushing		
Less than once a day	1	
Two or more times per day	2.200(1.725–2.806)	<0.001
6 Frequency of flossing		
Never	1	
Ever use	2.893(2.267–3.690)	<0.001
7 Frequency of rinsing mouth		
Never	1	
Ever use	1.922(1.532–2.412)	<0.001
8 Smoking		
Never	1	
Ever smoking	1.175(0.620–2.227)	0.621
9 Drink alcohol		
Never	1	
Ever smoking	1.015(0.787–1.308)	0.909
10 Do you pay close attention to pregnancy-related oral-health knowledge?		
No	1	
Yes	3.585(2.676–4.802)	<0.001
11 Do you have swelling and hyperplasia in gingiva during pregnancy?		
No	1	
Yes	1.124(.885–1.427)	0.337
12 Do you have tooth pain or peri-apical inflammation during pregnancy?		
No	1	
Yes	1.085(.821–1.433)	0.567
13 Are you satisfied with your oral health?		
Not satisfied	1	
Satisfied	1.341(1.065–1.688)	0.013
14 Are you satisfied with your individual dental hygiene behavior?		
Not satisfied	1	
Satisfied	2.213(1.763–2.778)	<0.001

‡ OR: Odds ratio.

§ CI: Confidence interval.

**Table 3:** The final model of multivariate logistic regression analysis to analyze the key factors underlying the routine utilization of dental care (RUDC) during pregnancy.

	Final Model	
Characteristics	OR‡ (95% CI§)	P value
1 Age		
30 or less	1	
31 or older	1.465 (1.127–1.903)	0.004
2 Annual household income		
Less than $8,000	1	
More than $8,000	1.958 (1.516–2.528)	<0.001
3 Frequency of tooth brushing		
Less than one time every day	1	
Two or more times per day	1.970 (1.516–2.561)	<0.001
4 Frequency of flossing		
Never	1	
Ever use	2.069 (1.580–2.708)	<0.001
5 Frequency of rinsing mouth		
Never	1	
Ever use	1.477 (1.145–1.904)	0.003
6 Do you pay close attention to pregnancy-related oral-health knowledge?		
No	1	
Yes	2.597 (1.900–3.549)	<0.001
7 Are you satisfied with your individual dental hygiene behavior?		
Not satisfied	1	
Satisfied	1.816 (1.417–2.327)	<0.001

‡ OR: Odds ratio.

§ CI: Confidence interval.

## Results

In total, 2259 pregnant women were included in this study (average age 29.28±3.38; mean±standard deviation). Most were aged between 21 and 35 (93.9%), had an educational level higher than high school (79.7%), and an annual household income of more than $4,500 (76.6%) (Table 1). While 65.64% paid for dental care through various insurances or were reimbursed by their employers, 32.89% had to pay for their dental care themselves.

Although 98.09% of the participants reported brushing at least once a day, 78.4% reported that they never used floss, and 59.7% that they never rinsed their mouth. A very small number of participants (0.44%) reported smoking during pregnancy, and 1.11% reported drinking alcohol. Nearly two-thirds of participants (62.06%) reported that “they paid close attention to oral-health knowledge during pregnancy”, while 31.83% reported swelling or hyperplasia in the gingiva during pregnancy, and 19.70% reported tooth pain or peri-apical inflammation during pregnancy. Only about 35% were very satisfied with their oral health and individual dental hygiene behavior.

In all, 16.73% (*n* = 367) of the participants reported that they had RUDC during pregnancy. The ages of the participants either with or without RUDC showed a normal distribution. Moreover, there was no significant age difference (*P*>0.05) between those who did and did not have RUDC. The percentage of pregnant women who have RUDC during pregnancy was positively correlated with their ages, educational levels and annual household incomes (Table 1).

The Univariate test found no significant influence for the following factors: “smoking” (*P* = 0.621), “drinking alcohol” (*P* = 0.909), “do you have swelling or hyperplasia in gingiva during pregnancy” (*P* = 0.337) and “do you have tooth pain or peri-apical inflammation during pregnancy” (*P* = 0.567) (Table 2). Consequently, these 4 factors were excluded for the following logistic regression analysis.

In the final model of multivariate logistic regression analysis, we identified 7 key underlying factors (Table 3). A significantly lower percentage of RUDC during pregnancy was associated with participants aged 30 or less or with annual income of less than $8,000. A significantly lower percentage was also found in participants who brushed their teeth once or less per day and in those who reported never using floss or rinsing their mouth. In addition, participants were more likely to have RUDC during pregnancy if they reported that they “paid close attention to pregnancy-related dental knowledge” and were “satisfied with their individual dental hygiene behavior”.

## Discussion

Improving pregnant women’s oral health by promoting their RUDC is one of the key means of safeguarding the health of their babies. Although the Chinese government has organized various public activities such as “Love Teeth Day” [9], China still has very few programs for promoting RUDC in pregnant women. To develop appropriate programs and policies, it is therefore of paramount importance to obtain the precise characteristics of the target population. To the best of our knowledge, this is the first study to analyze the current status and the key factors underlying RUDC in pregnant women in China.

While China has the world’s largest population, it has a weak tradition of RUDC. Most patients (51–75%) use dental care when they have acute problems or pain [10]. At 16.70%, the percentage of women using routine dental care during pregnancy was significantly lower than that in developed western countries such as the USA (30–50%) [11], [12]. Since Hangzhou is one of the richest cities in China, with a per-capita GDP (gross domestic product) of $12,447 in 2011, such a low percentage is unexpected. The situation may be even worse in China’s relatively less developed cities and rural areas; in the latter, the percentage of dental visits was found to be significantly lower than for urban residents [13]. Our data thus indicate a great need for policies and programs to encourage pregnant women in China to make dental visits.

Our data also highlight the importance of analyzing factors that may prevent these women from utilizing dental care. The reason for such low routine utilization is unclear. It cannot be attributed to the low incidence of oral diseases, since the incidence of “swelling and hyperplasia in gingiva” (*p* = 0.337) and “tooth pain or peri-apical inflammation” (*p* = 0.567) during pregnancy had no significant association with RUDC (Table 2). Meanwhile, a subjective factor–“Are you satisfied with your oral health?”–proved not to be a key underlying factor either. This data suggests that pregnant women without RUDC could not be simply seen in terms of a problem-oriented utilization pattern [14]. Interestingly, another subjective factor–“are you satisfied with your individual dental hygiene behavior?”–showed significant relevance (*P*<0.001) (Table 2), and proved to be a key underlying factor (*P*<0.001) (Table 3). In our study, however, we could not identify the causal relationship between this subjective factor and RUDC.

One of the factors most investigated for its influence on RUDC–not only in pregnant women but also in ordinary adults–is age [14]. We found that a pregnant woman’s age was indeed a key factor underlying her RUDC (Table 3): the percentage of such women who had RUDC showed an age-dependent increasing trend (Table 1), reaching 22.88% for those aged over 36. Those aged 30 or less were less likely to have RUDC than those aged 31 and older (Table 3). This may be due to their relatively lower age-related social and economic status. On the other hand, our results were not consistent with findings in USA showing that pregnant women aged over 36 were less likely to receive routine dental care than those aged 18–36 [15]. While further studies are needed to clarify the reasons, the disparity suggests that the age patterns related to RUDC differ between these two countries. We also found a significantly lower percentage of RUDC for pregnant women who brushed once or less per day and never used floss (Table 2). Similarly, we found that pregnant women had less routine dental care if they had to pay for their own dental care. These data suggested that the economically disadvantaged status was highly associated with a lower percentage of RUDC. Although a positive correlation was also detected between RUDC and educational level (Table 1), the final model of multivariate logistic regression analysis did not identify educational level as a key factor underlying RUDC. This result was consistent with earlier studies showing that educational level was not a key factor underlying pregnant women’s RUDC in the USA [15], [16].

Interestingly, we found that a significantly higher percentage of RUDC was associated with pregnant women who “paid close attention to pregnancy-related oral-health knowledge” (3 times higher) and “were satisfied with their individual dental hygiene behavior” (1.85 times higher) (Table 1). These two factors also proved to be an underlying key factor in the final multivariate logistic regression model (Table 3), thereby suggesting that pregnant women who really cared about their oral health were more likely to receive routine dental care.

One limitation of this study was our sampling method. We could not randomly and systematically select participants in the area of Hangzhou, as no information was available on pregnant women there. We therefore designed our sampling method on the basis of the social and medical situation in this city. In Hangzhou, all pregnant women have to be registered at the Department of Civil Affairs. They also have to take pregnancy courses, which are organized by general hospitals and by district-level centers. We therefore decided to incorporate our survey into the courses, which would give us a high response rate and better compliance. For this reason, we chose to conduct our study in one large general hospital and two small district healthcare centers.

Hangzhou City has are about ten large general hospitals. The largest-scale and most intensive pregnancy courses are provided by two of these hospitals: The Women’s Hospital, Medical College, Zhejiang University, and Sir Run Run Shaw Hospital, Medical College, Zhejiang University. We selected the former, as it is the largest and also the most specialized in women’s and children’s diseases. It is also very popular for the maternity care it provides.

Hangzhou City has five main districts: Shangcheng, Xiacheng, Gongshu, Jianggan and Xihu. For this study, we randomly chose the centers for Disease Prevention and Control and for Women and Children’s Healthcare in two of these districts, Gongshu and Jianggan. To check the representativeness of samples, we estimated the distribution of participants in all five districts by dividing the number of participants in one district by the total population in the same district. The results were as following: 0.63‰ in Shangcheng, 0.68‰ in Xiacheng, 0.47‰ in Gongshu, 0.51‰ in Jianggan, and 0.57‰ in Xihu. As this showed the distribution of the participants to be homogeneous throughout the five districts, our data are representative for the whole of Hangzhou City.

This type of hospital-based survey has in fact already been used previously in a study at the University of North California (UNC) Women’s Clinic Ultrasound Unit, which included women who were visiting the Women’s Clinic Ultrasound Unit for a clinically indicated prenatal ultrasound [15]. And this type of hospital-based survey in the three local hospitals had the advantage of a significantly higher response rate over letter surveys. In this study, data were obtained on the basis of a local survey that could not represent the situation in the whole of China. A nation-wide conclusion can be drawn only after conducting a systematic nation-wide survey.

In conclusion, our study found a very low rate of RUDC in pregnant woman in eastern China. If such women are to receive routine dental care and are thereby to improve their babies’ health, appropriate programs and policies are urgently needed.
